# Significance of Positive Cerebrospinal Fluid Cytology (Leptomeningeal Metastasis) in Central Nervous System Metastasis – A Multicentre Clinicopathological Review

**DOI:** 10.1111/pin.70142

**Published:** 2026-06-21

**Authors:** Hoi Wai Chan, Ka Man Cheung, Amy M. K. Chu, Sharon C. L. Ho, Chin Pan Kong, Joanna K. M. Ng, Hei Ming Lai, Peter W. T. Lee, Joshua J. X. Li, Chun Yan So, Gabriel C. H. Wong, Bryan C. W. Li

**Affiliations:** ^1^ Department of Medicine, Queen Mary Hospital The University of Hong Kong Pok Fu Lam Hong Kong SAR; ^2^ Department of Clinical Oncology Queen Elizabeth Hospital King's Park Hong Kong SAR; ^3^ Department of Clinical Oncology Pamela Youde Nethersole Eastern Hospital Chai Wan Hong Kong SAR; ^4^ Department of Clinical Oncology Tuen Mun Hospital Tuen Mun Hong Kong SAR; ^5^ Department of Pathology, Queen Mary Hospital The University of Hong Kong Pok Fu Lam Hong Kong SAR; ^6^ Department of Chemical Pathology, Prince of Wales Hospital The Chinese University of Hong Kong Pok Fu Lam Hong Kong SAR; ^7^ Department of Pathology Queen Elizabeth Hospital King's Park Hong Kong SAR; ^8^ Department of Clinical Oncology, Queen Mary Hospital The University of Hong Kong Pok Fu Lam Hong Kong SAR

**Keywords:** central nervous system metastasis, cerebrospinal fluid cytology, leptomeningeal metastasis

## Abstract

Leptomeningeal metastases (LM) is a rare but clinically important subtype in central nervous system (CNS) metastasis. Multicentre data was reviewed to detail the epidemiology, clinicopathological associations among patients with positive cerebrospinal fluid cytology (CSF) cytology. Patients with CNS metastasis and clinical data were identified through the Hong Kong Hospital Authority Clinical Data Analysis and Reporting System (CDARS). In total, through 1st January 2000 to 31st December 2024, 38,893 patients with CNS metastasis were retrieved, 1503 had CSF cytology. The most common primary malignancies were lung (54.16%), breast (11.18%) and gastrointestinal (5.66%; colonic: 3.33%, gastric: 1.86%, esophageal: 0.47%). There were 220 (14.64%) patients with positive CSF, they were younger (57.72 years vs. 60.03, *p* = 0.005), and likely with breast (20.84%, *p* = 0.016) and lung primaries (16.47%, *p* = 0.030). The median overall survival (OS) was 113 days. Positive CSF, age > 60 and gastrointestinal primary were correlated with shorter OS (*p* < 0.05). The inclusion of brain radiotherapy (260 day vs. 82, *p* < 0.001) and systemic therapy (496 days vs. 61, *p* < 0.001) for treatment of leptomeningeal metastases was independently associated with longer OS. Positive CSF cytology is a strong independent indicator of poor outcomes. Radiotherapy and systemic therapy improve overall survival for the medium term (6–12 months).

## Graphical abstract

Positive CSF cytology is a strong independent indicator of poor outcomes in CNS metastasis, along with advanced age and primary gastrointestinal malignancy. Radiotherapy and systemic therapy improve overall survival in CNS metastasis for the medium term (6–12 months), including those with LM.

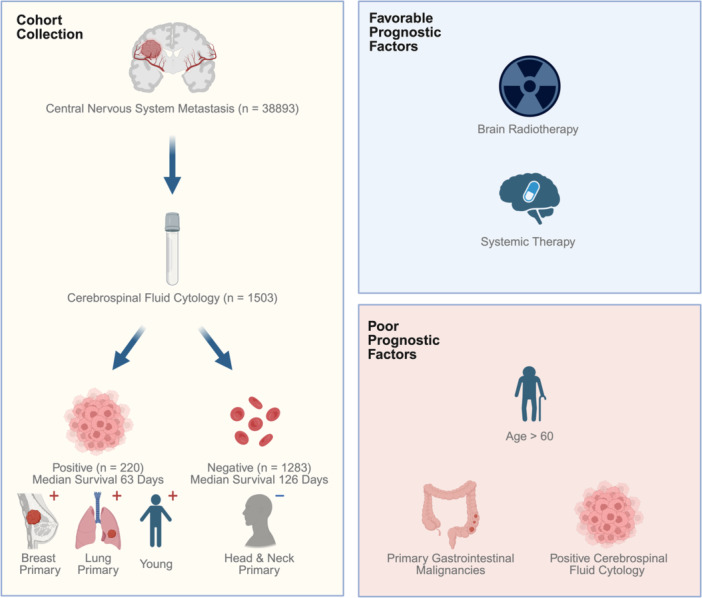

## 1 Introduction

Leptomeningeal involvement is a rare yet serious event in the disease course of central nervous system metastasis [1]. Leptomeningeal metastasis is diagnosed by magnetic resonance imaging and/or cerebrospinal fluid cytology. The diagnostic performances and clinical utility of both modalities are complimentary, with overall satisfactory performance while not without limitations [2, 3]. Cerebrospinal fluid cytology is robust in terms of achieving a tissue diagnosis [4], and a positive diagnosis in cerebrospinal fluid can be considered as a reference positive, which is an important finding necessitating prompt clinical action and meticulous oncology and palliative care planning [5]. In this study, a retrospective Hong Kong‐wide database review was performed to identify patients with central nervous system metastasis and cerebrospinal fluid cytology obtained for detailing the epidemiology, clinicopathological associations with prognosis and benefit of radiotherapy in this uncommon condition, aiming to provide clinical insights in oncological management following a positive cerebrospinal fluid cytology diagnosis.

## 2 Methodology

Patients of age 18 years of above with the ICD‐9‐CM code 198.3 (secondary malignant neoplasm of the brain/spine) were identified through the Hong Kong Hospital Authority Clinical Data Analysis and Reporting System (CDARS) through the period 1st January 2000 to 31st December 2024. Clinicopathological information of the identified patients were obtained through a reference key unique to each patient. Other parameters retrieved included demographical data (age and sex), registered death information (updated to October 2025), latest follow‐up attendance (updated to October 2025), other diagnostic codes in addition to 198.3, radiotherapy treatment records, medication dispensation (Supporting Information S1) and pathology laboratory information (cerebrospinal fluid cytology diagnosis). Overall survival was calculated from the first diagnosis of brain metastasis to death or last follow‐up date (alive). Cases without matched cerebrospinal fluid cytology were excluded. Patients records included those from the Hospitals of the 5 involved hospital clusters (Hong Kong East Cluster, Hong Kong West Cluster, Kowloon Central Cluster, New Territories East Cluster and New Territories West Cluster) including 35 hospitals and institutions.

Cerebrospinal fluid cytology with a diagnosis of carcinoma, malignancy or equivalent are considered as positive. Indeterminate and haematolymphoid cytologic diagnoses were not considered positive. If multiple cerebrospinal fluid cytology entries were present, any positive diagnosis was considered as positive. Site of primary malignancy was determined from review of the diagnostic codes of each patient. Statistical analysis was performed using SPSS (version 26.0) and R. The t‐test and Chi‐square test were used for comparison of age, sex and site of primary malignancy with cerebrospinal fluid cytology diagnosis as continuous and categorical variables. Survival analysis was performed using the Kaplan‐Meier method. Multivariate analysis was performed using Cox proportional hazard model with backward variable selection using Wald method. A *p*‐value of < 0.05 was considered significant.

## 3 Results

A total of 38,893 patients with central nervous system metastasis were retrieved, and 1503 had a matching of cerebrospinal fluid cytology specimen and corresponding cytologic diagnosis. The most common sites of primary malignancy were lung (*n* = 814/1503, 54.16%), breast (*n* = 168/1503, 11.18%), gastrointestinal (*n* = 85/1503, 5.66%) and head and neck (*n* = 44/1503, 2.93%). These four populated groups were used for further survival analysis. The detailed composition of site of primary, including the remaining cases, is detailed in Supporting Information Table S1. There were 220 (14.64%) patients with a positive cerebrospinal fluid cytology and 1283 without any positive diagnoses, from 751 male and 752 female patients with an average age of 59.66 (18–92) years.

Patients with a positive cerebrospinal fluid cytology were younger in age (57.72 vs. 60.03, *p* = 0.005), and more frequently occurring in patients with breast (20.84%, *p* = 0.016) and lung malignancies (16.47%, *p* = 0.030), while rarely presenting in those with primary head and neck malignancies (2.28%, *p* = 0.019) (Table 1).

**Table 1:** Cerebrospinal fluid (CSF) cytology diagnosis and site of primary malignancy.

	CSF negative	CSF positive	Total	*p*‐value
Breast	133 (79.17%)	35 (20.84%)	168	0.016
Gastrointestinal	68 (80%)	17 (20%)	85	0.150
Head and neck	43 (97.73%)	1 (2.28%)	44	0.019
Lung	680 (83.54%)	134 (16.47%)	814	0.030
Multiple	110 (88.71%)	14 (11.3%)	124	0.270
Others	249 (92.92%)	19 (7.09%)	268	
	1283 (85.37%)	220 (14.64%)	1503	

**Figure 1:**
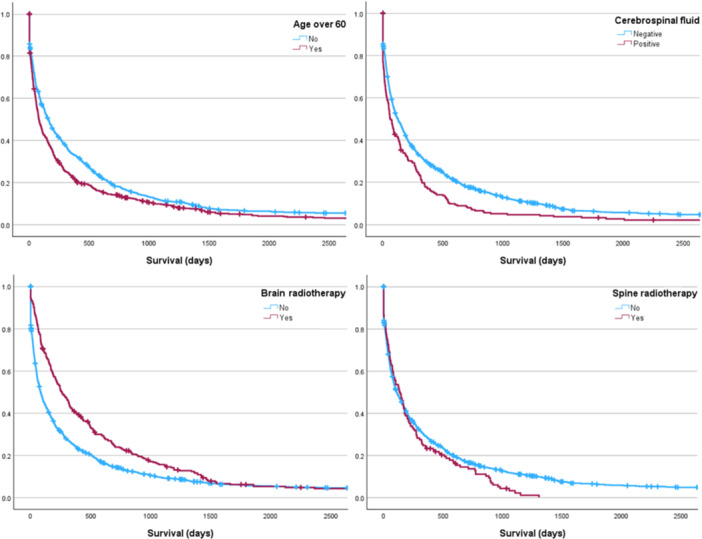
Overall survival by (a) age, (b) cerebrospinal fluid cytology, and (c, d) history of radiotherapy. The overall survival was worse for a/top left patients age over 60 years (*p* < 0.001) and b/top right patients with positive cerebrospinal fluid (*p* < 0.001). c/bottom left brain radiotherapy improved overall survival (*p* < 0.001) while the difference in overall survival was not significant for d/bottom right) spine radiotherapy (*p* = 0.125).

The median OS of the cohort was 113 days. Kaplan Meier analysis revealed association between shorter OS and age over 60 years (median 79 days vs. 157 days, *p* < 0.001) and positive cerebrospinal fluid cytology (median 63 days vs. 126 days, *p* < 0.001), and longer OS for patients who received brain radiotherapy (260 days vs. 82 days, *p* < 0.001) and systemic therapy (496 days vs. 61 days, *p* < 0.001) (Table 2). Similar significant survival differences were also observed with subgroup analysis limited to patients with positive cerebrospinal fluid (age over 60 years: median 45 days vs. 102 days, *p* = 0.031; brain radiotherapy: median 282 days vs. 49 days, *p* = 0.002). Survival advantage for patients receiving brain radiotherapy was demonstrated up to 1 year in those who had negative cerebrospinal fluid cytology, and up to 6 months in those positive (Table 3) (Figure 1).

**Table 2:** Survival analyzes.

	Median survival	95% confidence interval	*p*‐value (univariate)	*p*‐value (multivariate)
Age				
≤ 60	157	129.006–184.994		
> 60	79	64.453–93.547	< 0.001	0.003
Sex				
Male	117	90.387–143.613		
Female	108	85.105–130.895	0.210	0.099
Primary site				
Breast	90	45.919–134.081		
Gastrointestinal	54	17.302–90.698		
Head and neck	69	19.162–118.838		
Lung	136	111.320–160.680		
Multiple	208	91.843–324.157		
Others	85	44.538–125.462	< 0.001	< 0.001
Cerebrospinal fluid cytology				
Negative	126	105.751–146.249		
Positive	63	38.357–87.643	< 0.001	< 0.001
Brain radiotherapy				
Not received	82	67.730–96.270		
Received	260	206.145–313.855	< 0.001	< 0.001
Spine radiotherapy				
Not received	108	89.842–126.158		
Received	133	84.227–181.773	0.125	0.037
Systemic therapy				
Not received	61	52.338–69.662		
Received	496	434.030–557.970	< 0.001	< 0.001

**Table 3:** Comparison of survival between patients with (a) negative and (b) positive cerebrospinal fluid (CSF) cytology.

(a)	Brain RT	No Brain RT	Odds ratio	(b)	Brain RT	No brain RT	Odds ratio
3‐month survival							
Alive	203	516	2.526		24	77	3.060
Deceased	76	488	(1.890–3.380)		11	108	(1.415–6.617)
6‐month survival							
Alive	166	401	2.209		21	58	3.284
Deceased	113	603	(1.686–2.895)		14	127	(1.561–6.913)
1‐year survival							
Alive	118	230	1.895		10	34	1.776
Deceased	161	724	(1.440–2.494)		25	151	(0.781–4.043)
5‐year survival							
Alive	25	102	0.870		4	10	2.258
Deceased	254	902	(0.550–1.377)		31	175	(0.666–7.655)

**Figure 2:**
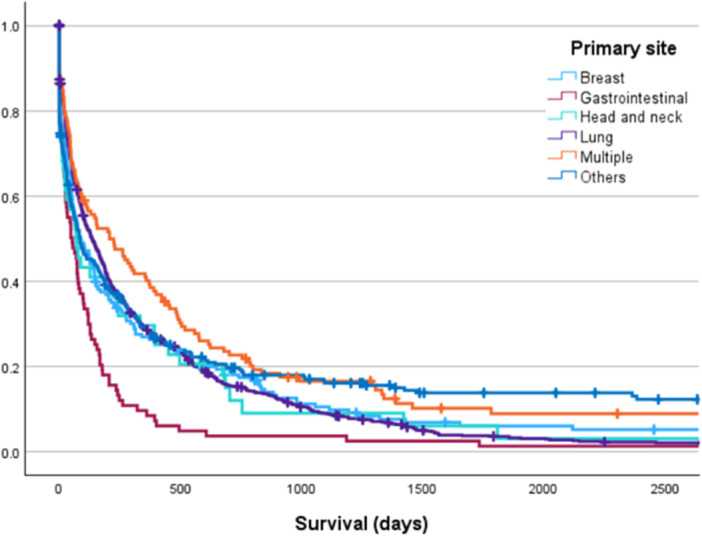
Site of primary and overall survival.

OS for patients with gastrointestinal (median 54 days vs. 123 days, *p* < 0.001) was significantly worse, the difference in other sites of primary malignancy did not reach statistical significance (*p* > 0.05). In patients with positive cerebrospinal fluid cytology, those with primary gastrointestinal malignancies also performed the worst (median 25 days vs. 75 days, *p* = 0.012), however, the median OS for primary lung malignancies was better (median 94 days vs. 25 days, *p* = 0.004) (Figure 2). Cox regression revealed that all associations on univariate analysis ‐ age over 60 years (*p* = 0.003), positive cerebrospinal fluid cytology (*p* < 0.001), site of primary (*p* < 0.001), brain radiotherapy (*p* < 0.001) and systemic therapy (*p* < 0.001) remained independently significant. History of spine radiotherapy was associated with shorter OS (*p* = 0.037).

## 4 Discussion

**Figure 3:**
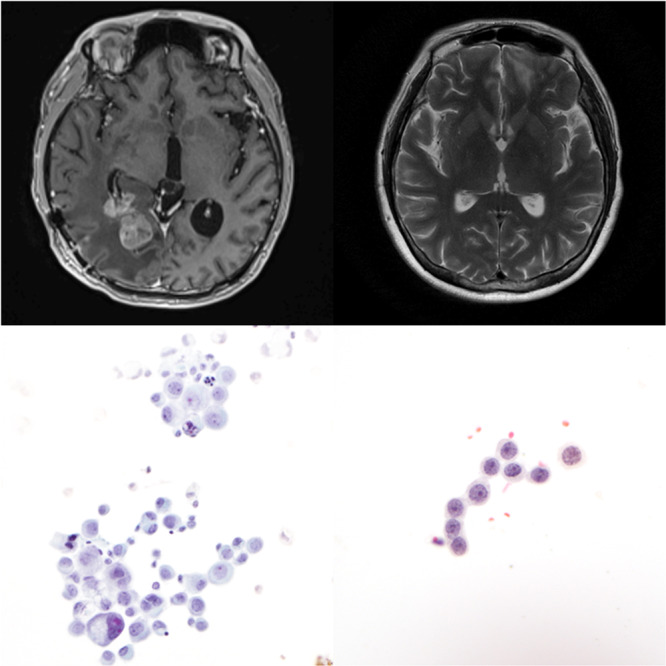
Radiological and cytological examples of leptomeningeal metastasis. Magnetic resonance imaging showing (a/top left) metastatic lung carcinoma at the right temporo‐occipital lobe with midline shift and abutting the cisterns and (b/top right) metastatic breast carcinoma presenting as an extra‐axial nodule at inferior left frontal lobe. Cerebrospinal fluid cytology showing (c/bottom left) pleomorphic vacuolated malignant cells in metastatic lung carcinoma and (d/bottom right) a hyperchromatic cluster of tumor cells in metastatic breast carcinoma.

The outcomes of patients with central nervous system metastasis is poor, as in our cohort the median OS was less than 4 months, similar to that reported in the literature [6]. The survival of patients with cytology‐proven leptomeningeal metastasis is even shorter, as reflected by a median OS of 63 days with a positive cerebrospinal fluid cytology, half of the median survival compared to those without (162 days). Cerebrospinal fluid cytology is the reference tissue diagnostic modality for the diagnosis of leptomeningeal metastasis [7] (Figure 3). Although magnetic resonance imaging (MRI) are more commonly used in detection of leptomeningeal metastasis due to its non‐invasiveness [3], cerebrospinal fluid cytology remains unchallenged in terms of positive predictive value and the availability of tissue material for ancillary testing, notably immunocytochemistry and sequencing [8, 9]. Regardless, both diagnostic modalities are similar in sensitivity and considered as complementary [10]. Owing to the lack of any superior reference standard compared to cerebrospinal fluid cytology and MRI, any positive diagnostic result from either or both cytology or magnetic resonance imaging is an indication for further clinical decision.

Similar to brain metastasis [11], breast (20.84%) and lung (16.47%) are the sites of primary most frequently involving the leptomeningeal space. The distribution corresponds to the high incidence of breast and lung cancers and may not necessarily indicate an increased propensity of leptomeningeal spread over other primaries. The gastrointestinal malignancies were the third most frequent and demonstrated worst OS overall (any central nervous system metastasis) or in those with positive cerebrospinal fluid cytology. Melanoma has also been reported, in Caucasian populations, to be a common source of leptomeningeal spread [12, 13], but is less seen in Asian populations, particularly non tropical regions such as Hong Kong [14]. Breast and lung carcinomas on metastasis have an arsenal of active systemic therapies including hormonal treatments, targeted treatments including HER2, EGFR, ALK and ROS1 inhibitors, which are known to have good penetration to the blood‐brain barrier [15, 16, 17], and immunotherapies. Whereas the current therapeutic options for gastrointestinal malignancies are more limited, the advent of novel treatment targets including claudin 18.2 antibody‐drug conjugates and combined‐immunotherapeutic approaches holds the promise of improving survival [18].

Historically, the blood‐brain barrier is an obstacle for systemic therapy in treatment of central nervous system disease [19], and localized therapy is often deployed in parallel, including intrathecal agents, which are high in toxicity and modest in benefit [20, 21], surgery and radiotherapy. As compared to surgical resection, which is often opted for fit patients with small number but symptomatic large volume metastasis [22], radiotherapy has been the mainstay in treatment of most central nervous system metastasis [23, 24]. Our data has demonstrated that both systemic therapy and brain radiotherapy is an independent favorable prognostic factor, along with poor prognostic factors of older age, positive cerebrospinal fluid cytology and metastasis from primary gastrointestinal malignancies. The survival benefit is seen in patients with leptomeningeal metastasis for up to 6 months.

Patients receiving spinal radiotherapy had a shorter OS compared to those without. This is likely explained by the confounding factor of spinal metastasis but not the radiotherapy resulting in a reduction in survival. Spinal metastasis and associated neurological deficits can lead to life threatening complications such as respiratory depression, bedsores, deep vein thrombosis, urinary tract infection and pneumonia, contributing to a shortened survival [25, 26]. At the time point of 3‐month survival, for patients with positive cerebrospinal fluid cytology, 76% (*n* = 13/17) of patients who received radiotherapy were alive, whereas 43% (*n* = 88/203) were deceased, giving an odds ratio of 4.247 (95% C.I. 1.339–13.474), indicating a significant positive effect for spinal radiotherapy for short term survival. In comparison, the odds ratio for the group of patients with negative cerebrospinal fluid cytology was 0.963 (95% C.I. 0.664–1.396) and was not significant, as was for both cytology positive and negative groups at longer follow‐up time points, indicating only survival benefit within the short term.

With regards to limitations, the current study is conducted on a population‐wide database, individual patient data such as the radiotherapy treatment volume and severity of the leptomeningeal involvement are unavailable. Notably, two clinically relevant parameters – tumor subtype and performance status were not retrievable. For breast and lung cancers, tumor grade and histological type are prognostically significant parameters on diagnosis and recurrence [27, 28]. Performance status has demonstrated strong correlation with survival in patients with brain metastasis notwithstanding the short baseline survival of this patient group [29], and it remains uncertain whether age would still show independent statistical significance in the current study if performance status was adjusted for.

The shortcomings of the current study, in terms of above confounding factors, is compensated by the large case number and availability of pathology results which ensures the reliability of diagnosis. Data on newer generations of targeted therapies demonstrating strong intracranial therapy, notably Osimertinib for lung cancer [30] and Tucatinib for breast cancer [31], were not available in the current cohort. Regardless, systemic therapy has shown survival benefit, and the use of these agents is expected to further improve the treatment outcomes of patients with central nervous system and/or leptomeningeal metastasis.

## 5 Conclusion

Our territory‐wide real‐world data suggests that radiotherapy is associated with better short‐term survival in patients with leptomeningeal metastasis, controlled for age, primary site and use of systemic treatments. Selection of fit and suitable patients remains key to ensure risk‐benefit balance for maximum benefit.

## Author Contributions

HWC contributed to conceptualization, methodology, data curation, investigation, resources, and project administration. KMC contributed to conceptualization, data curation, investigation, validation, and manuscript review and editing. AMKC and SCLH contributed to conceptualization, data curation, and investigation. CPK contributed to conceptualization, data curation, investigation, and resources. JKMN contributed to writing the original draft, data curation, investigation, and resources. HML contributed to data curation and resources. PWTL contributed to validation. JJXL contributed to writing the original draft, conceptualization, data curation, formal analysis, methodology, investigation, resources, and project administration. CYS and GCHW contributed to conceptualization, data curation, investigation, validation, and manuscript review and editing. BCWL contributed to conceptualization, data curation, investigation, methodology, resources, supervision, and validation.

## Funding

The authors have nothing to report.

## Ethics Statement

Ethical approval was granted by the Hospital Authority Central Institutional Review Board (reference number: CIRB‐2025‐387‐2), Joint Chinese University of Hong Kong—New Territories East Cluster Clinical Research Ethics Committee (reference number 2025.582) and The University of Hong Kong/Hospital Authority Hong Kong West Cluster Institutional Review Board (reference number: UW 25‐423, 24‐292) with waiver of the requirement written informed consent. The research has been performed in accordance with the Declaration of Helsinki.

## Conflicts of Interest

The authors declare no conflicts of interest.

## Supporting information

Supporting File 1

Supporting File 2

## Data Availability

The data that support the findings of this study are available from the corresponding author upon reasonable request.
