# Supplementary material for: Significance of Positive Cerebrospinal Fluid Cytology (Leptomeningeal Metastasis) in Central Nervous System Metastasis – A Multicentre Clinicopathological Review
Source: Pathol Int. 2026 Jun 21;76(6):e70142. doi: 10.1111/pin.70142 (PMC13283487; doi:10.1111/pin.70142)
Supplement: Supplementary file 1 — Supporting File 1 [file PIN-76-0-s001.docx]

Supplementary material 1. Drug list for systemic therapy in the cohort

IFOSFAMIDE

ACTINOMYCIN D

BENDAMUSTINE

BLEOMYCIN

BUSULFAN

CARMUSTINE

CARBOPLATIN

CISPLATIN

CYCLOPHOSPHAMIDE

CYTARABINE

DAUNORUBICIN

DOCETAXEL

DOXORUBICIN

EPIRUBICIN

ERIBULIN

ETOPOSIDE

FLUDARABINE

FLUOROURACIL

GEMCITABINE

IRINOTECAN

LOMUSTINE

MELPHALAN

METHOTREXATE

MITOMYCIN

MITOXANTRONE

PACLITAXEL

PEMETREXED

PROCARBAZINE

VINBLASTINE

VINCRISTINE

VINORELBINE

AFINITOR / EVEROLIMUS

ALECTINIB

BEVACIZUMAB

CETUXIMAB

CRIZOTINIB

ERLOTINIB

GEFITINIB

IMATINIB MESYLATE

LAPATINIB

NERATINIB

PERTUZUMAB

RITUXIMAB

SORAFENIB

TRASTUZUMAB

* All drug dispensary entries were reviewed
