# Supplementary material for: Significance of Positive Cerebrospinal Fluid Cytology (Leptomeningeal Metastasis) in Central Nervous System Metastasis – A Multicentre Clinicopathological Review
Source: Pathol Int. 2026 Jun 21;76(6):e70142. doi: 10.1111/pin.70142 (PMC13283487; doi:10.1111/pin.70142)
Supplement: Supplementary file 2 — Supporting File 2 [file PIN-76-0-s002.docx]

Supplementary table 1. Site of primary malignancy of patients in the cohort with cerebrospinal fluid (CSF) cytology diagnosis

|  | CSF negative | CSF positive |  |
| --- | --- | --- | --- |
| Bladder | 4 | 0 | 4 |
| Breast | 133 | 35 | 168 |
| Colon | 44 | 6 | 50 |
| Esophagus | 7 | 0 | 7 |
| Head and neck | 43 | 1 | 44 |
| Kidney | 13 | 0 | 13 |
| Liver | 12 | 0 | 12 |
| Lung | 680 | 134 | 814 |
| Melanoma | 5 | 1 | 6 |
| Multiple | 110 | 14 | 124 |
| Ovary | 4 | 1 | 5 |
| Pancreas | 7 | 0 | 7 |
| Peritoneum | 1 | 0 | 1 |
| Prostate | 7 | 0 | 7 |
| Skin | 2 | 0 | 2 |
| Stomach | 17 | 11 | 28 |
| Testis | 3 | 0 | 3 |
| Thymus | 5 | 0 | 5 |
| Thyroid | 7 | 0 | 7 |
| Uterine cervix | 5 | 1 | 6 |
| Unknown | 172 | 16 | 188 |
| Uterus | 2 | 0 | 2 |
|  | 1283 | 220 | 1503 |
